# Synthesis of 1,2,3-Triazole Derivatives and *in Vitro* Antifungal Evaluation on *Candida* Strains

**DOI:** 10.3390/molecules17055882

**Published:** 2012-05-16

**Authors:** Reginaldo G. Lima-Neto, Nery N. M. Cavalcante, Rajendra M. Srivastava, Francisco J. B. Mendonça Junior, Almir G. Wanderley, Rejane P. Neves, Janaína V. dos Anjos

**Affiliations:** 1 de Micologia Médica, Centro de Ciências Biológicas, Universidade Federal de Pernambuco (UFPE), 50670-901, Recife, PE, Brazil; 2 de Síntese Orgânica, Departamento de Química Fundamental, Universidade Federal de Pernambuco (UFPE), 50740-560, Recife, PE, Brazil; 3 de Síntese e Vetorização de Substâncias Bioativas, Universidade Estadual da Paraíba (UEPB), 58058-420, João Pessoa, PB, Brazil; 4 de Farmacologia Pré-Clínica e Toxicologia de Produtos Bioativos, Departamento de Fisiologia e Farmacologia, Universidade Federal de Pernambuco (UFPE), 50670-901, Recife, PE, Brazil

**Keywords:** click chemistry, 1,2,3-triazoles, *Candida* spp., antifungal activity

## Abstract

1,2,3-Triazoles have been extensively studied as compounds possessing important biological activities. In this work, we describe the synthesis of ten 2-(1-aryl-1*H*-1,2,3-triazol-4-yl)propan-2-ols via copper catalyzed azide alkyne cycloaddition (CuAAc or *click chemistry*). Next the*in vitro* antifungal activity of these ten compounds was evaluated using the microdilution broth method against 42 isolates of four different *Candida* species. Among all tested compounds, the halogen substituted triazole 2-[1-(4-chlorophenyl)-1*H*-(1,2,3)triazol-4-yl]propan-2-ol, revealed the best antifungal profile, showing that further modifications could be done in the structure to obtain a better drug candidate in the future.

## 1. Introduction

Deep and superficial fungal infections have increased significantly over the past few decades. Control of fungal disease has proved to be difficult because of several risk factors. The number of patients at highest risk for these infections has been steadily increasing, especially among patients immunocompromised due to AIDS, organ transplantation, chemotherapy or other invasive procedures [1]. Because of this, there is a clear need for the development of effective antimycotic therapeutic agents for the treatment of fungal infections, since the major classes of antifungal drugs available have encountered resistance in clinical use [2,3]. Among these classes, azoles are the most used because of their broad spectrum, high potency and low toxicity [4].

Azoles are competitive inhibitors of lanosterol 14 α-demethylase (a cytochrome P-450 enzyme), leading to a decrease in the fungal biosynthesis of ergosterol, which is a key compound of fungal cell membranes, thereby preventing fungal growth [5,6]. Beyond the antifungal properties [7,8,9], triazoles possess a variety of interesting biological activities, forming part of the scaffolds of antibacterial and antituberculosis agents [10,11,12,13,14], neuraminidase inhibitors [15], anticancer compounds [16], antiviral agents [17], analgesic compounds [18], herbicides [19] and plant growth regulators [20].

Considering the above mentioned advantages of triazole-containing antifungal drugs and the increasing drug resistance mechanisms in these type of microorganisms, we decided to synthesize 2-(1-aryl-1*H*-1,2,3-triazol-4-yl)propan-2-ols capable of inhibiting cell growth of some *Candida* species with clinical relevance and testing their activity using the microdilution broth method.

## 2. Results and Discussion

### 2.1. Chemistry

**Scheme 1:**
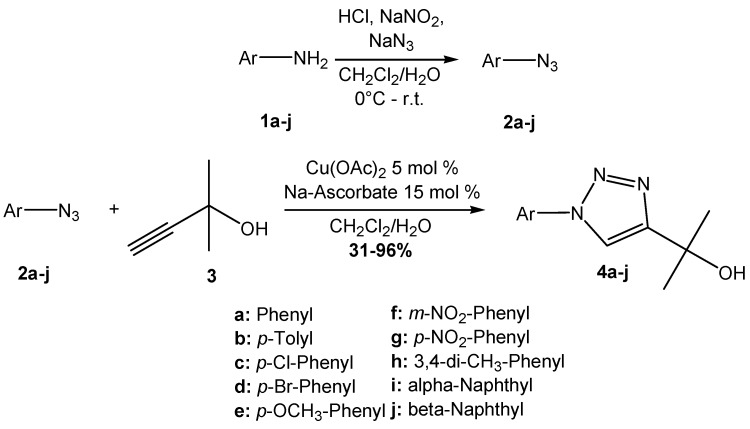
Synthesis of triazoles **4a**–**j**.

Prior to the synthesis of the 2-(1-aryl-1*H*-1,2,3-triazol-4-yl)propan-2-ols, the aromatic azides **2a**–**j** were prepared from the corresponding anilines **1a**–**j** following the Sandmeyer conditions [21]. The aromatic azides were then reacted with 2-methylbut-3-yn-2-ol (**3**) using Cu(OAc)_2_ and sodium ascorbate as catalyst in 1:1 dichloromethane:water [22,23] to give the products **4a**–**j** in good yields (Scheme 1).

For all synthesized molecules, only one regioisomer could be detected by ^1^H-NMR. Only one singlet was observed in the ^1^H-NMR spectrum for the triazole ring (δ 7.19–8.16 ppm), which can be attributed to the proton in the C-5 position of the triazole nucleus. According to earlier literature on copper catalyzed cycloadditions [24,25], it is believed that the obtained products are 1,4-regioisomers. 

### 2.2. Biology

For each experiment, inocula controls produced clearly detectable growth after the chosen incubation period, indicating that all isolates were viable and that the conditions used were suitable for fungal growth. The antifungal screening results by MIC measurements are summarized in Table 1. Most of the synthesized 1,2,3-triazoles showed weak (**4a**,**b**,**d**,**e**,**f**) or no activity (**4g**,**h**,**i**,**j**) against the *Candida* species used herein. However, for 2-[1-(4-chlorophenyl)-1*H*-(1,2,3)triazol-4-yl]propan-2-ol (**4c**) and for the reference drug (fluconazole), it was possible to determine a MIC for *Candida* growth. As it can be seen, fluconazole showed fungistatic activity in concentrations ranging from 0.5 to 64 μg·mL^−1^. Eight isolates (4987, 4986, 4984, 4970, 4790, 4608, 1059 and 109) had their growth inhibited in a dose-dependent pattern, presenting MIC values ranging from 16 to 32 μg·mL^−1^. In contrast to these findings, nine isolates (4990, 4802, 4388, 4263, 4261, 4124, 3719, 1150 and 934) were resistant to the reference azole showing MIC values above 64 μg·mL^−1^. It can be also observed that the triazole **4c** showed good antifungal activity, presenting MIC values ranging from 64 to 256 μg·mL^−1^ against all the tested strains. 

**Table 1:** Antifungal activity of triazoles **4a**–**j** and fluconazole against the studied *Candida* strains.

Tested strain n° URM	Compounds (MICs in μg·mL^−1^) ^a^										
	4a	4b	4c	4d	4e	4f	4g	4h	4i	4j	Flu ^b^
4990	2,048	1,024	256	2,048	1,024	2,048	R	R	R	R	>64
4987	2,048	1,024	128	2,048	1,024	2,048	R	R	R	R	16
4986	2,048	1,024	128	2,048	1,024	2,048	R	R	R	R	16
4820	2,048	1,024	256	2,048	1,024	2,048	R	R	R	R	4
4819	2,048	1,024	128	1,024	1,024	2,048	R	R	R	R	4
4817	2,048	1,024	128	1,024	1,024	2,048	R	R	R	R	4
4609	2,048	1,024	128	1,024	1,024	2,048	R	R	R	R	4
4606	2,048	1,024	128	1,024	1,024	2,048	R	R	R	R	2
4388	2,048	1,024	128	1,024	1,024	2,048	R	R	R	R	>64
4387	2,048	1,024	128	1,024	1,024	2,048	R	R	R	R	2
4386	2,048	1,024	128	2,048	1,024	2,048	R	R	R	R	1
4385	2,048	1,024	128	1,024	1,024	2,048	R	R	R	R	2
4384	2,048	1,024	128	1,024	1,024	2,048	R	R	R	R	2
4260	2,048	1,024	128	1,024	1,024	2,048	R	R	R	R	2
4127	R	1,024	128	1,024	2,048	2,048	R	R	R	R	0.5
4126	2,048	1,024	128	1,024	1,024	2,048	R	R	R	R	0.5
4125	2,048	1,024	256	1,024	1,024	2,048	R	R	R	R	0.5
4124	2,048	1,024	256	2,048	1,024	2,048	R	R	R	R	>64
3719	R	1,024	256	2,048	1,024	2,048	R	R	R	R	64
3716	R	1,024	256	2,048	1,024	2,048	R	R	R	R	0.5
4802	2,048	2,048	256	2,048	1,024	R	R	R	R	R	64
4263	2,048	2,048	256	2,048	1,024	R	R	R	R	R	64
1059	2,048	1,024	64	512	1,024	2,048	R	R	R	R	16
934	2,048	2,048	256	2,048	1,024	R	R	R	R	R	64
109	2,048	1,024	128	1,024	1,024	2,048	R	R	R	R	16
4984	R	2,048	128	1,024	1,024	2,048	R	R	R	R	16
4970	R	2,048	128	1,024	512	2,048	R	R	R	R	16
4889	R	1,024	64	1,024	1,024	2,048	R	R	R	R	2
4818	R	2,048	128	1,024	1,024	2,048	R	R	R	R	4
4804	R	2,048	128	1,024	1,024	2,048	R	R	R	R	8
4608	R	1,024	128	1,024	1,024	2,048	R	R	R	R	16
4607	R	1,024	128	1,024	1,024	2,048	R	R	R	R	4
4261	R	2,048	256	1,024	1,024	R	R	R	R	R	>64
3627	2,048	1,024	64	1,024	1,024	2,048	R	R	R	R	0.5
3624	2,048	1,024	64	1,024	1,024	2,048	R	R	R	R	0.5
3621	2,048	1,024	64	1,024	1,024	2,048	R	R	R	R	0.5
22019 ^c^	R	2,048	256	2,048	1,024	2,048	R	R	R	R	8
4790	R	2,048	128	2,048	1,024	2,048	R	R	R	R	32
4262	R	1,024	128	2,048	1,024	2,048	R	R	R	R	4
1150	R	2,048	128	2,048	1,024	2,048	R	R	R	R	>64
933	R	2,048	128	2,048	1,024	2,048	R	R	R	R	4
916	R	2,048	128	2,048	1,024	2,048	R	R	R	R	4

^a^ The MIC value was defined as the lowest concentration of the antifungal agent and were read after two days at 37 °C. Inocula sizes contained approximately 2.5 × 10^3^ cells·mL^−1^. Culture media tested were the RPMI 1640 (Sigma Chemical Co., St. Louis, MO, USA). The final concentration of triazoles was between 4–2,048 μg·mL^−1^ and 0.125–64 μg·mL^−1^ for fluconazole; ^b^ Fluconazole; ^c^ *Candida parapsilosis* ATCC 22019 was used as reference strain. R = Resistance.

Observing the drug structure, it can be noticed that **4c** possesses a chlorine atom substituted in the *para* position of the phenyl ring present at the *N*-1 position of the triazole nucleus. Chlorine-substituted rings were found to be good antifungal tools, as reported by Wang and colleagues [26]. In their study, sixteen *N*-methyl-substituted phenoxybutan-1-amine chloro-substituted derivatives exhibited strong *in vitro* antifungal activity, being more active against the tested microorganisms than the used reference drug, voriconazole. Later, Wang *et al.* [27] decided to synthesize fourteen novel triazole-substituted compounds containing a phenoxyalkyl group. They also observed that the best antifungal drugs were those with halogen atoms as substituents in the phenyl rings.

The acute preliminary toxicological tests in rats showed that the oral administration of triazole **4c** at the 2,000 mg·kg^−1^ dose did not produce any signs of toxicity or mortality, indicating that the lethal dose for 50% of the animal population in this study (LD_50_) is above 2,000 mg·kg^−1^. According to Lorke [28], substances presenting a LD_50 _higher than 2,000 mg·kg^−1^ can be considered low toxicity drugs. Since our pharmacological studies have shown that **4c** is active in 64 to 256 μg·mL^−1^ concentrations, those toxicological findings demonstrate that this drug candidate is quite safe for further *in vivo* studies and can be considered an an antifungal lead for this class of compounds.

## 3. Experimental

### 3.1. General

All commercially available reagents were used without any further purification and the reactions were monitored by TLC analysis (TLC plates GF_254_ E. Merck). Melting points were determined on a Büchi apparatus and are uncorrected. Column chromatography was performed on Silica Gel 60 (70–230 mesh, Merck Chemicals International). NMR spectra were recorded with a Bruker AC-200 MHz spectrometer (Billerica, MA, USA) and referenced as follows: ^1^H (200 MHz), internal SiMe_4_ at δ = 0.00 ppm, ^13^C (50 MHz), internal standard at δ = 77.23 ppm. Exact mass measurements of the molecular ions were obtained on a Shimadzu LC/MS-IT-TOF Eletrospray.

### 3.2. Synthesis of the Aromatic Azides ***2a–j***

To a solution of the corresponding aniline **1a**–**j** (4.1 mmol) dissolved in CH_2_Cl_2_ (30 mL), was added 6 N HCl (30 mL) at 0 °C. To this biphasic system was added dropwise a saturated aqueous solution of NaNO_2_ (10 mL). After stirring for 30 min at 0 °C, NaN_3_ (0.53 g, 8.2 mmol) was added at 0 °C. Stirring was maintained for 30 min, and the mixture was allowed to warm to room temperature. The two phases were separated, and the aqueous phase was extracted with CH_2_Cl_2_ (3 × 20 mL). The combined organic layers were washed with aqueous solution of NaHCO_3_, then brine, dried (Na_2_SO_4_) and filtered from active charcoal. Evaporation of the solvent *in vacuo* gave the crude azides **2a**–**j** that were used in the next step without further purification.

### 3.3. Synthesis of 2-(1-Aryl-1H-1,2,3triazol-4-yl)propan-2-ols ***4a–j***

2-Methylbut-3-yn-2-ol (**3**, 1,1 mmol) and the azido compound **2a**–**j** (1 mmol) were suspended in a 1:1 mixture of CH_2_Cl_2_ and water (10 mL). To this solution was added a mixture of Cu(OAc)_2_ (36 mg, 0.2 mmol) and sodium ascorbate (79 mg, 0.4 mmol). The resulting mixture was stirred at room temperature until TLC analysis indicated complete consumption of the azide. The mixture was diluted with CH_2_Cl_2_ (5 mL) and water (5 mL). The organic layer was separated, and the water phase was extracted again with CH_2_Cl_2_ (5 mL). The combined organic layers were dried over Na_2_SO_4_. Removal of the solvent *in vacuo* gave a residue that was recrystallized from chloroform-hexanes to afford the corresponding triazoles **4a**–**j**. 

*2-(1-Phenyl-1H-1*,*2*,*3 -triazol-4-yl)propan-2-ol* (**4a**): White crystals; yield 86%; m.p.: 95–96 °C; *R*_f_ 0,60 (ethyl acetate-chloroform 9:1, v/v). ^1^H-NMR (CDCl_3_): δ 0.97 (6H, s); 2.92 (1H, bs); 6.73 (3H, m); 6.93 (1H, dd, *J* = 8 Hz, 2 Hz); 6.97 (1H, dd, *J* = 8 Hz, 2 Hz); 7.24 (1H, s). ^13^C-NMR (CDCl_3_): δ 30.3; 68.5; 117.7; 120.4; 128.5; 129.6; 136.9; 156.4. ESI–HRMS *m/z*: 226.0911 (calcd. for C_11_H_13_N_3_ONa [M+Na]^+^: 226.0956). 

*2-[1-(4-Tolyl)-1H-1*,*2*,*3 -triazol-4-yl]propan-2-ol* (**4b**): White crystals; yield 78%; m.p.: 120–121 °C; *R*_f_ 0,65 (ethyl acetate-chloroform 9:1, v/v). ^1^H-NMR (CDCl_3_): δ 0.99 (6H, s); 1.68 (3H, s); 3.13 (1H, bs); 6.54 (2H, bd, *J* = 8.0 Hz); 6.85 (2H, bd, *J* = 8.0 Hz); 7.24 (1H, s). ^13^C-NMR (CDCl_3_): δ 20.9; 30.4; 68.4; 117.7; 120.2; 130.0; 134.6; 138.5; 156.2. ESI–HRMS *m/z*: 240.1049 (calcd. for C_12_H_15_N_3_ONa [M+Na]^+^: 240.1113). 

*2-[1-(4-Chlorophenyl)-1H-1*,*2*,*3 -triazol-4-yl]propan-2-ol* (**4c**): White crystals; yield 67%; m.p.: 93–94 °C; *R*_f_ 0,67 (ethyl acetate-chloroform 9:1, v/v). ^1^H-NMR (CDCl_3_): δ 0.98 (6H, s); 2.90 (1H, bs); 6.75 (2H, dd, *J* = 8.0 Hz, 2 Hz); 6.93 (2H, dd, *J* = 8.0 Hz, 2.0 Hz); 7.24 (1H, s). ^13^C-NMR (CDCl_3_): δ 30.3; 68.5; 107.1; 117.6; 121.5; 129.7; 134.2; 135.4; 156.6. ESI–HRMS *m/z*: 260.0518 (calcd. for C_11_H_12_ClN_3_ONa [M+Na]^+^: 260,0567). 

*2-[1-(4-Bromophenyl)-1H-1,2,3-triazol-4-yl]propan-2-ol* (**4d**): White crystals; yield 96%; m.p.: 95–96 °C; *R*_f_ 0,68 (ethyl acetate-chloroform 9:1, v/v). ^1^H-NMR (CDCl_3_): δ 1.70 (6H, s); 3.68 (1H, bs); 7.59 (4H, m); 7.97 (1H, s). ^13^C-NMR (CDCl_3_): δ 30.3; 68.4; 107.0; 121.7; 122.1; 132.7; 135.8; 156.7. ESI–HRMS *m/z*: 305.9989 (calcd. for C_11_H_12_BrN_3_ONa [M+Na]^+^: 304.0061). 

*2-[1-(4-Methoxyphenyl)-1H-1*,*2*,*3 -triazol-4-yl]propan-2-ol* (**4e**): Red crystals; yield 69%; m.p.: 106–107 °C; *R*_f_ 0,65 (ethyl acetate-chloroform 9:1, v/v). ^1^H-NMR (CDCl_3_): δ 1.67 (6H, s); 3.00 (1H, bs); 3.82 (3H, s); 6.96 (2H, dd, *J* = 6.0 Hz, 4.0 Hz); 7.57 (2H, dd *J* = 6.0 Hz, 4.0 Hz); 7.81 (1H, s). ^13^C-NMR (CDCl_3_): δ 30.4; 55.5; 68.5; 114.6; 122.1; 130.5; 159.6. ESI–HRMS *m/z*: 238.0757 (calcd. for C_12_H_13_ClNO_2_ [M+H]^+^: 238.0635). 

*2-[1-(3-Nitrophenyl)-1H-1*,*2*,*3 -triazol-4-yl]propan-2-ol* (**4f**): White crystals; yield 75%; m.p.: 98–100 °C; *R*_f_ 0,60 (ethyl acetate-chloroform 9:1, v/v). ^1^H-NMR (CDCl_3_): δ 1.63 (6H, s); 3.96 (1H, bs); 7.65 (1H, bd, *J =* 8.0 Hz);8.12 (2H, m); 8.16 (1H, s); 7.65 (1H, d, *J =* 2.1 Hz). ^13^C-NMR (CDCl_3_): δ 30.2; 68.4; 114.9; 122.9; 125.8; 130.8; 137.5; 148.6; 157.1. ESI–HRMS *m/z*: 271.0764 (calcd. for C_11_H_12_N_4_O_3_Na [M+Na]^+^: 271.0807). 

*2-[1-(4-Nitrophenyl)-1H-1*,*2*,*3 -triazol-4-yl]propan-2-ol* (**4g**): Yellow crystals; yield 58%; m.p.: 123–124 °C; *R*_f_ 0,63 (ethyl acetate-chloroform 9:1, v/v). ^1^H-NMR (CDCl_3_): δ 1.69 (6H, s); 2.82 (1H, bs); 7.95 (2H, dd, *J =* 6.0 Hz, 4.0 Hz); 8.03 (1H, s); 8.37 (2H, dd, *J =* 6.0 Hz, 2.0 Hz). ^13^C-NMR (CDCl_3_): δ 30.4; 68.7; 107.1; 120.3; 125.5; 141.2; 147.0; 157.2. ESI–HRMS *m/z*: 265.1469 (calcd. for C_11_H_13_N_4_O_4_ [M+H_2_O−H]^+^: 265.2453). 

*2-[1-(3,4-Dimethylphenyl)-1H-1*,*2*,*3-triazol-4-yl]propan-2-ol* (**4h**): yellow crystals; yield 31%; m.p.: 128–129 °C; *R*_f_ 0,80 (ethyl acetate:chloroform 9:1, v/v). ^1^H-NMR (CDCl_3_): δ 1.00 (6H, s); 1.60 (6H, s); 2.69 (1H, bs); 6.53 (1H, m);6.72 (2H, dd, *J =* 8.1 Hz, 2.0 Hz); 7.19 (1H, s). ^13^C-NMR (CDCl_3_): δ 19.3; 19.8; 30.4; 68.5; 107.1; 117.7; 121.5; 130.4; 134.9; 137.2; 138.1; 156.0. ESI–HRMS *m/z*: 254.1229 (Calcd for C_13_H_17_N_3_ONa [M+Na]^+^: 254.1269). 

*2-[1-( α -Naphthyl)-1H-1*,*2*,*3-triazol-4-yl]propan-2-ol* (**4i**): Red crystals; yield 62%; m.p.: 152–153 °C; *R*_f_ 0,65 (ethyl acetate:chloroform 9:1, v/v). ^1^H-NMR (CDCl_3_): δ 1.75 (6H, s); 3.00 (1H, bs); 7.54 (4H, m); 7.82 (1H, s); 7.95 (3H, m). ^13^C-NMR (CDCl_3_): δ 30.5; 68.6; 122.3; 123.5; 127.0; 128.2; 128.5; 130.3; 133.7; 134.0; 155.5. ESI–HRMS *m/z*: 276.1070 (calcd. for C_15_H_15_N_3_ONa [M+Na]^+^: 276.1113). 

*2-[1-( β -Naphthyl)-1H-1*,*2*,*3-triazol-4-yl]propan-2-ol* (**4j**): Yellow crystals; yield 60%; m.p.: 143–144 °C; *R*_f_ 0,68 (ethyl acetate:chloroform 9:1, v/v). ^1^H-NMR (CDCl_3_): δ 1.72 (6H, s); 2.97 (1H, bs); 7.53 (2H, d, *J* *=* 8.1 Hz); 7.89 (4H, m); 8.03 (1H, s); 8.12 (1H, d, *J* *=* 2.1 Hz). ^13^C-NMR (CDCl_3_): δ 30.4; 68.6; 118.3; 118.9; 126.9; 127.3; 127.8; 128.2; 129.9; 132.7; 133.1; 134.4; 155.6. ESI–HRMS *m/z*: 276.1071 (calcd. for C_15_H_15_N_3_ONa [M+Na]^+^: 276.1113).

### 3.4. Strains and Growth Cultures

Twenty strains of *Candida albicans*, five of *Candida krusei*, eleven of *Candida parapsilosis* and five of *Candida tropicalis* were supplied by the URM Culture Collection of the Department of Mycology, Biological Sciences Centre of the Federal University of Pernambuco, Recife, Brazil. Strains have been stocked in mineral oil at 18 °C [28]. Viability tests and subsequent taxonomic confirmation of their morphological, biochemical and physiological characteristics were carried out [29]. Species, accession numbers, stock time and isolation substratum are summarized in Table 2. 

**Table 2:** Samples of *Candida* species preserved in the Mycotheca Culture Collection—University of Recife Mycology (URM).

Species	Accession nº (URM)	Storage (years)	Substratum
*C. albicans*	4990	01	Vaginal secretion
*C. albicans*	4987	01	Vaginal secretion
*C. albicans*	4986	01	Vaginal secretion
*C. albicans*	4820	02	Ungual scrap
*C. albicans*	4819	02	Ungual scrap
*C. albicans*	4817	02	Ungual scrap
*C. albicans*	4609	03	Blood
*C. albicans*	4606	03	Blood
*C. albicans*	4388	05	Oropharyngeal secretion
*C. albicans*	4387	05	Oropharyngeal secretion
*C. albicans*	4386	05	Oropharyngeal secretion
*C. albicans*	4385	05	Oropharyngeal secretion
*C. albicans*	4384	05	Oropharyngeal secretion
*C. albicans*	4260	05	Oropharyngeal secretion
*C. albicans*	4127	07	Inguinal area
*C. albicans*	4126	07	Urine
*C. albicans*	4125	07	Spittle
*C. albicans*	4124	07	Oropharyngeal secretion
*C. albicans*	3719	10	Tooth scrap
*C. albicans*	3716	10	Tooth scrap
*C. krusei*	4802	02	*
*C. krusei*	4263	05	Oropharyngeal secretion
*C. krusei*	1059	48	*
*C. krusei*	934	49	Appendix biopsy
*C. krusei*	109	52	*
*C. parapsilosis*	4984	01	Vaginal secretion
*C. parapsilosis*	4970	01	Vaginal secretion
*C. parapsilosis*	4889	02	Blood
*C. parapsilosis*	4818	02	Ungual scrap
*C. parapsilosis*	4804	02	IFM
*C. parapsilosis*	4608	03	Blood
*C. parapsilosis*	4607	03	Blood
*C. parapsilosis*	4261	05	Oropharyngeal secretion
*C. parapsilosis*	3627	12	Spittle
*C. parapsilosis*	3624	12	Spittle
*C. parapsilosis*	3621	12	Spittle
*C. parapsilosis*	ATCC22019	-	-
*C. tropicalis*	4790	02	Cassava powdery
*C. tropicalis*	4262	06	Oropharyngeal secretion
*C. tropicalis*	1150	46	Tongue
*C. tropicalis*	933	49	Vaginal secretion
*C. tropicalis*	916	49	Feces

* Substratum not identified.

### 3.5. *In vitro* Antifungal Susceptibility

Reference microdilution trays, containing serial drug dilutions were prepared by following the CLSI M27-A3 guidelines [30]. The triazoles were dissolved in dimethylsulfoxide (DMSO) and then these stock solutions were stored at −80 °C. The concentrations tested ranged from 2 to 2,048 μg·mL^−1^. Fluconazole was used as reference drug at concentrations from 0.125 to 64 μg·mL^−1^. In order to obtain a fungal inoculum containing 1–5 × 10^6^ CFU·mL^−1^, each strain was cultured on a tube containing 20 mL of Sabouraud Dextrose Agar (SDA) plus yeast extract at 35 °C for two days. After this time, yeast suspensions were prepared in sterile physiological solution (0.85%) and maintained at 28 ± 2 °C and then were adjusted to 90% transmittance at 530 nm. Two serial dilutions from 1:100 and 1:20 sequentially were made to obtain a final inoculum containing 1.0 × 10^3^ and 5 × 10^3^ CFU·mL^−1^. The microdilution wells containing 100 µL of the twofold serial dilutions of the test and reference drugs in standard RPMI 1640 medium (Sigma Chemical Co., St. Louis, MO, USA), buffered to pH 7.0 with 0.165 mol·L^−1^ of morpholinopropanesulphonic acid (MOPS, Sigma), were inoculated with 100 µL of inoculum. After inoculation, the microplates were incubated at 35 °C in a non CO_2_ incubator and were read visually 48 h after the incubation. MICs corresponded to the lowest drug dilution that showed growth inhibition compared to untreated yeasts. *C. parapsilosis* ATCC 22019 was used as reference strain. All tests wereperformed in triplicate.

### 3.6. Animals and Preliminary Toxicological Tests

Adult male Wistar rats (*Rattus norvegicus*), aged 2–3 months, weighing 220–260 g, were obtained from the Pound of the Department of Physiology and Pharmacology at the Federal University of Pernambuco. They were kept under standard environmental conditions (23 ± 2 °C; 12:12 h dark/light cycle) and water and animal food (Labina^®^, Purina, Brazil) were made available *ad libitum*. The animals were randomly divided into two groups (*n* = 3–4/group) and deprived of feed for 12 h with access to water *ad libitum*. Further, group 1 received vehicle (solution of 2.5% tween 80) and group 2 received 2-[1-(4-chlorophenyl)-1H-1,2,3-triazol-4-yl]propan-2-ol (4c) in a single oral dose of 2,000 mg·kg−1. The observations were performed at 30, 60, 120, 180 and 240 min after the oral treatments and then, daily for 14 days. Behavioral changes, weight, consumption of food and water, clinical signs of toxicity, and mortality were recorded daily [31]. The experimental protocol was approved by the Federal University of Pernambuco’s Ethics Committee for Animal Experimentation (Process n^o^ 23076.003830). Studies of acute toxicity were performed according to “Up and down” method with slight modifications, as described by OECD 425 [32].

## 4. Conclusions

In conclusion, a series of analogs of 1,2,3-triazoles with ten distinct substituents at the *N-*1 of the triazole ring were synthesized and assessed for their antifungal activity. All compounds were tested against 42 pathogenic strains of four different *Candida* species. Modification of substituents has a great impact on the minimal inhibitory concentration values, since we could obtain triazole derivatives showing no antimycotic activity, with moderate antifungal activity and one compound with promising activity. The antifungal tests data show that the chloro-substituted triazole derivative exhibited, in particular, good fungal growth inhibition, showing that further modifications in the 2-(1-aryl-1*H*-1,2,3-triazol-4-yl) series can be done in order to obtain more potent prototypes.
